# Prevalence and Characteristics of People with Disabilities Among Abused Victims in Saudi Arabia

**DOI:** 10.1007/s44197-024-00252-2

**Published:** 2024-06-05

**Authors:** Abdulaziz S. Alangari, Duaa Alammari, Norah Alhowaish, Waseemah Almutairi, Zainab Alnjeidi, Majid Aleissa

**Affiliations:** 1 Department of Epidemiology and Biostatistics, College of Public Health and Health Informatics, King Saud Bin Abdulaziz University for Health Sciences, Riyadh, Saudi Arabia; 2 King Abdullah International Medical Research Center, Riyadh, Saudi Arabia; 3 Department of Health Systems Management, College of Public Health and Health Informatics, King Saud Bin Abdulaziz University for Health Sciences, Riyadh, Saudi Arabia; 4 King Saud Bin Abdulaziz University for Health Sciences, Riyadh, Saudi Arabia; 5 National Family Safety Program, King Abdulaziz Medical City, Ministry of the National Guard-Health Affairs, Riyadh, Saudi Arabia

**Keywords:** Abuse, Violence, Disability, Public health, Saudi Arabia

## Abstract

**Background:**

Abuse is an ongoing public health issue that results in increased morbidity and mortality rates. Abuse against individuals with disabilities is a pervasive and deeply concerning issue, often compounded by factors of vulnerability and dependence. The majority of disabled individuals experience abuse, with the majority enduring it repeatedly. Identifying the problem is the first step towards preventing abuse. This study aimed to identify the prevalence of people with disabilities among abused victims and the victim’s associated risk factors in Saudi Arabia.

**Methods:**

This cross-sectional study obtained data from the National Family Safety Registry. All registered children and adults between April 2017 and December 2022 from 93 centers across 13 regions of Saudi Arabia were included. Logistic regression models were used to determine the association between independent variables and victim-related risk factors such as the onset of abuse complications, the victim being an adult or child, the victim’s gender, and whether they had been previously abused.

**Results:**

Individuals with disabilities comprise 1.4% (*n* = 199) of all reported cases of abuse (*n* = 14,004), and the trend of violence against people with disabilities has decreased during the 6-year study period. Of the abused people with disabilities, 72.4% were children, 57.8% were males, 45.2% were previously abused, and 65.3% had complications from the abuse. Caregiver type, perpetrator gender, perpetrator age, and previous abuse status were significant factors.

**Conclusions:**

This study highlights the disability prevalence among reported abuse cases and evaluates victim’s risk factors in Saudi Arabia, which demonstrates an urgency for targeted intervention and support. People with disabilities constitute a vulnerable demographic who require increased support and resources. Comprehensive data collection can be utilized for effective violence prevention strategies. Further research should explore qualitative methods and survey the rates of abuse among people with disabilities in the community to gain deeper insights.

## Introduction

Violence is a major global public health concern. More than 1.3 million individuals are estimated globally lose their lives each year due to acts of violence, and nearly 25% of children and 30% of women worldwide experience physical or sexual violence at some point in their lives [1]. In addition, interpersonal violence is among the top five causes of disability-adjusted life years (DALYs) among adolescents aged 10–24 years [2]. One study estimated that 15.7% of people aged 60 years and older have been abused [3]. This violence issue is recognized internationally. The United Nations includes violence in its 17 Sustainable Development Goals (SDGs) for 2030 [4]. SDGs 16.1 and 16.2 are key components of the broader agenda for promoting peace, justice, and strong institutions. It specifically targets a reduction in all forms of violence and related deaths worldwide, including violence against children. Recognizing that violence in its various manifestations undermines social cohesion, impedes development, and violates human rights, these goals call for concerted efforts to address this pervasive challenge and to create safer and more secure environments for all individuals.

Approximately 16% of the world’s population suffers from some kind of disability and rates have risen in the past decade [5] primarily due to an increase in aging populations and chronic diseases. People with disabilities have a 50% increased risk of violence compared to those without disabilities because of their need for continuous support, inability to defend themselves, and reduced communication abilities [6–10]. According to a national survey in the United States, 70% of people with disabilities have been exposed to abuse, and 90% have been abused on multiple occasions [11]. Verbal/emotional abuse was the most common form against people with disabilities (87.2%), followed by physical (50.6%), sexual (41.6%), neglect (37.3%), and financial (31.5%). In some studies, disability was a predictor for violence in females, especially with severe disability or impairment [12, 13]. In addition, disability is associated with an increased risk of sexual violence for both genders [14]. Children with disabilities are prone to all forms of abuse, especially those living in disadvantaged populations [15].

In Saudi Arabia, the Ministry of Health conducted the “2019 World Health Survey-Saudi Arabia” to aid policymakers in creating and overseeing initiatives and health strategies. In the survey, approximately 3% of women had experienced physical violence [16]. Different results were obtained in a systematic review that concluded one in every three women has been a victim of interpersonal violence [17]. Another study estimated among children a prevalence of 10% for sexual abuse and 65% for psychological abuse, which are affected by the caregiver type and gender of the victim [18]. The prevalence of people with disabilities was 7.1% in 2017 [19]. Little is known about the characteristics of abuse against people with disabilities worldwide or in Saudi Arabia. This study aimed to identify the prevalence of people with disabilities among abuse victims and the victim’s associated risk factors in Saudi Arabia.

## Methods

### Study Design and Participants

This cross-sectional study obtained data from the National Family Safety Registry (NFSR). The NFSR is a national hospital-based registry and a platform for research data regarding child and adult maltreatment and domestic violence in Saudi Arabia. According to the Saudi Law of Protection from Abuse [20], abuse encompasses any type of exploitation, such as physical, psychological, and sexual harm, threat of such harm, and negligence.

All registered children and adults with disabilities and evidence of abuse in the NFSR between April 2017 and December 2022 were included (*n* = 199). The sample of the NFSR varies over time but includes data from a significant number of cases reported across the country, ensuring a representative overview of violence issues in Saudi Arabia. Among the 270 registered centers in the NFSR, 93 were active and had reported cases. The cases were registered in secondary and tertiary hospitals across all 13 regions of Saudi Arabia. The hospitals have a multidisciplinary team (physicians, social workers, psychologists, and legal personnel) who are responsible for the diagnosis and management of abuse and neglect cases. The healthcare workers document the incidences using a standardized form of reporting that contains information about abuse.

Ethical approval for this study was obtained from the King Abdullah International Medical Research Center (#NRC23R-022-01, Feb 2023).

### Measures

Data were collected according to a predetermined list of variables designed by the NFSP. The data collection form included the following items:

1. *Victim sociodemographic information* age, gender, residence, caregivers, marital status, educational level, incident reporter (family member, victim, stranger, or healthcare worker), and employment status.
2. *Abuse information* specific information about the history and nature of the abuse; victim’s health condition before the abuse, nature of previous abuse (if any), nature of current abuse (physical, sexual, emotional; and neglect), and final status of the victim (no change, new disability, death, deterioration of health status, and psychiatric disorder).
3. *Alleged abuser information* if the abuser was known, sociodemographic information about the abuser and health information are explored. This included the relationship with the victim, gender, age, marital status, education level, employment status, and health conditions (chronic illness, disability, or addiction).
4. *Hospitalization and final decision* this stage evaluates the final disposition of the victim. The healthcare practitioner records whether the victim required hospital admission, discharge status, and referrals to the social protection committee or police.

### Statistical Methods

Descriptive statistics and frequency tables were used to describe participant characteristics. Frequencies and percentages are used for categorical variables, and means with standard deviations are used for continuous variables. The prevalence of people with disabilities was calculated based on the proportion of people with disabilities from the total abuse cases for each year. Logistic regression models were used to determine the association between independent variables and victim-related risk factors such as the onset of abuse complications, the victim being an adult or child, the victim’s gender, and whether they had been previously abused. The independent variables were caregiver type (both parents, one parent, or other), perpetrator gender, perpetrator age, and prior instances of abuse. Significance was estimated at a 2-tailed *p*-value of < 0.05, with a 95% confidence interval. All tests were conducted using SAS version 9.4 (SAS Institute Inc., Cary, NC, USA).

## Results

### Victim Characteristics

**Fig. 1:**
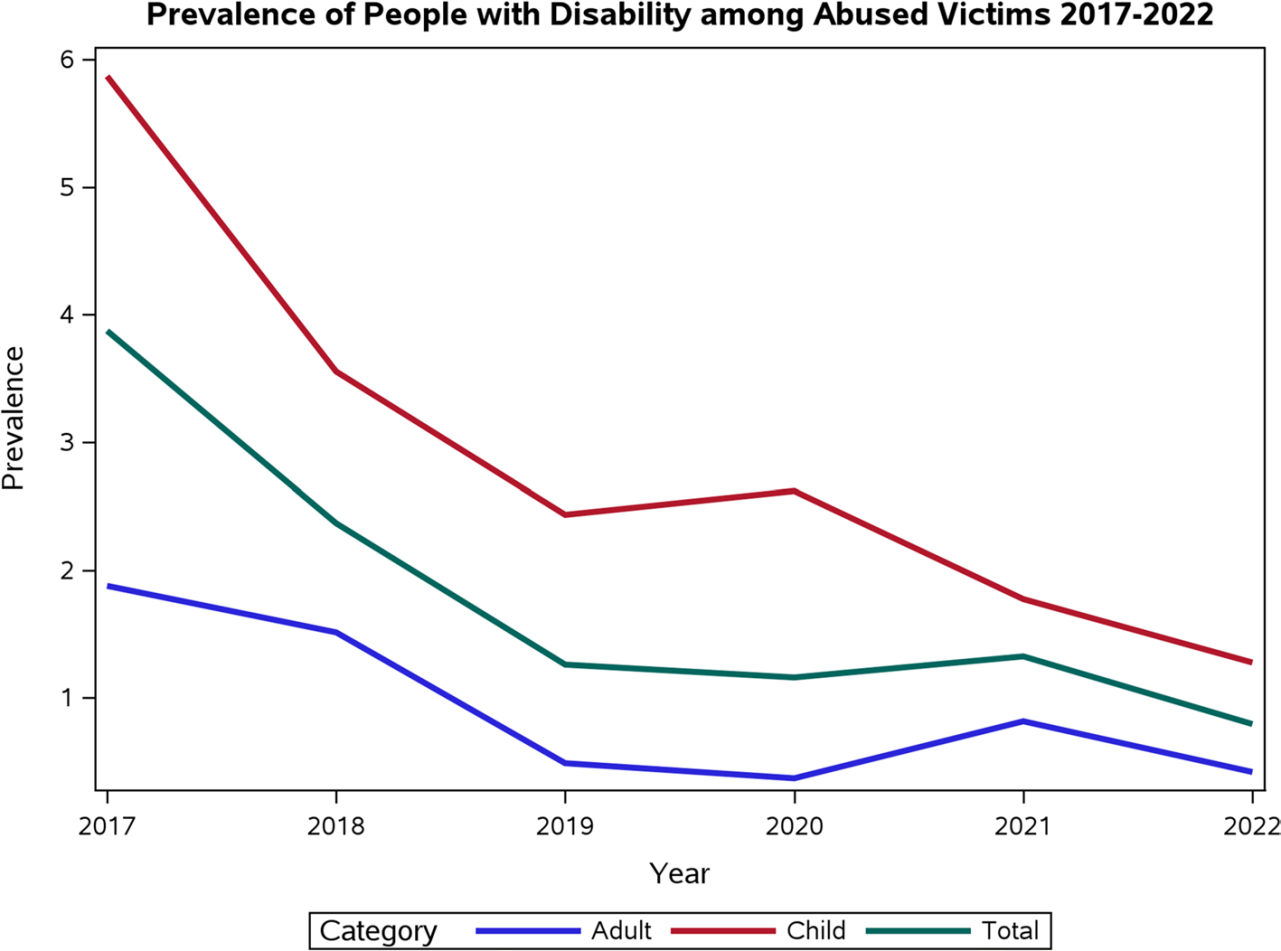
Prevalence of people with disability among abused victims 2017–2022

The total number of confirmed abuse incidents was 14,004. Of these, 199 were among people with disabilities (1.4%), of which 144 were children (72.4%) and 55 were adults (27.6%). Figure 1 presents the prevalence trend, which shows an overall decline between 2017 (3.9%) and 2022 (0.8%). The average age of these participants was 16.8 years (± 19.6) years; 7 years (± 4.1) years for child victims, and 42.4 (± 21.0) years for adult victims (Table 1). Among the abused children, the majority were males (62.5%), cared for by both parents (73.6%), and lived in either the Eastern (35.4%) or the Riyadh region (31.9%). The majority of abused adults were females (54.5%), cared for by others (36.4%), and lived in the Eastern (27.3%), Jizan (27.3%), or Riyadh region (23.6%). Moreover, most abused adults were single (50.9%), and about a third were married (32.7%). A substantial proportion of adults were illiterate (43.6%), followed by those with a high school or equivalent education (21.8%). Regarding employment status, 36.4% were unemployed and 27.3% were housewives.

**Table 1:** Victim characteristics (n = 199)

Variable	Children		Adults		All	
	Mean	± SD	Mean	± SD	Mean	± SD
Age	7.0	4.1	42.4	21.0	16.8	19.6
Gender	n	%	n	%	n	%
Male	90	62.5	25	45.5	115	57.8
Female	54	37.5	30	54.5	84	42.2
Caregivers						
Both parents	106	73.6	14	25.5	120	60.3
One parent	32	22.2	8	14.6	40	20.1
Husband/wife	0	0.0	13	23.6	13	6.5
Other	6	4.2	20	36.4	26	13.1
Region						
Riyadh Region	46	31.9	13	23.6	59	29.7
Eastern Region	51	35.4	15	27.3	66	33.2
Asir Region	5	3.5	1	1.8	6	3.0
Mecca Region	21	14.6	5	9.1	26	13.1
Jizan Region	9	6.3	15	27.3	24	12.1
Al-Bahah Region	1	0.7	1	1.8	2	1.0
Al-Qassim Region	8	5.6	1	1.8	9	4.5
Medina Region	2	1.4	2	3.6	4	2.0
Najran region	1	0.7	2	3.6	3	1.5
Reporter						
The victim	0	0.0	14	25.5	14	7.0
Family member	31	21.5	7	12.7	38	19.1
Health institution member	113	78.5	33	60.0	146	73.4
Strange person	0	0.0	1	1.8	1	0.5
Marital status	NA				NA	
Single			28	50.9		
Married			18	32.7		
Divorced			3	5.5		
Widowed			6	10.9		
Educational level	NA				NA	
Illiterate			24	43.6		
Elementary			7	12.7		
Intermediate			4	7.3		
High school or equivalent			12	21.8		
University or equivalent			4	7.3		
Unknown			4	7.3		
Employment	NA				NA	
Employed			3	5.5		
Student			2	3.6		
Freelancers			2	3.6		
Housewife			15	27.3		
Retired			7	12.7		
Unemployed			20	36.4		
Unknown			6	10.9		

*SD* standard deviation, *NA* not applicable

### Abuse Information

Nearly one-quarter (23.6%) of the abused children and nearly one-seventh (14.6%) of the abused adults had chronic diseases before abuse (Table 2). The most common form of abuse was neglect, all participants 59.3%, children 61.8%, and adults 52.7%; followed by physical abuse, all participants 42.2%, children 39.6%, and adults 49.1%. Among all forms of abuse, approximately one-third of the victims had no change in their final health status; all participants (34.7%, children 34.1%, and adults 36.3%. Similarly, health status deterioration was the second most common outcome; all participants 33.7%, children 29.2%, and adults 45.4%. Psychiatric disorders were the third most common outcome from abuse; all participants 11.1%, children 11.9%, and adults 9.1%. Among all victims, 45.2% had been previously abused; children 44.4%, and adults 47.3%. Of those, neglect accounted for 26.6% of them: 27.1% of children and 25.5% of adults. This was followed by physical abuse; all of them 19.1%, children 16.7%, and adults 25.5%.

**Table 2:** Abuse information (n = 199)

	Children		Adults		All	
	n	%	n	%	n	%
Victim’s health conditions before abuse						
Chronic illness	34	23.6	8	14.6	42	21.1
No	110	76.4	47	85.5	157	78.9
Previous abuse						
Yes	64	44.4	26	47.3	90	45.2
No	50	34.7	12	21.8	62	31.2
Unknown	30	20.8	17	30.9	47	23.6
Nature of previous abuse^a^						
Physical	24	16.7	14	25.5	38	19.1
Sexual	2	1.4	0	0.0	2	1.0
Neglect	39	27.1	14	25.5	53	26.6
Emotional	10	9.1	5	6.9	15	7.5
Nature of current abuse^a^						
Physical	57	39.6	27	49.1	84	42.2
Sexual	6	4.2	7	12.7	13	6.5
Neglect	89	61.8	29	52.7	118	59.3
Emotional	19	13.2	12	21.8	31	15.6
Victim final status						
No change	49	34.1	20	36.3	69	34.7
New disability	5	3.5	0	0.0	5	2.5
Dead	3	1.8	1	2.1	4	2.0
Deterioration of health status	42	29.2	25	45.4	67	33.7
Psychiatric disorder	17	11.9	5	9.1	22	11.1
Unknown	28	19.5	4	7.1	32	16.1

^a^Percentages cannot be added up because of multiple selections

### Perpetrator Information

Information on the abusers was available for 178 abuse incidents (Table 3). The most common abusers of children were fathers (35.4%), followed by mothers (33.1%) and both parents (20.0%). In contrast, the majority of those who abused adults were those other than parents (79.2%), followed by fathers (14.6%). Males were the most common abusers (all participants 50.0%, children 40.8%, and adults 75.0%) followed by females (all participants 34.8%, children 39.2%, and adults 22.9%). The most common age group for abusers was 31–40 years (40.0% of children abusers and 35.4% of adult abusers) followed by 41–50 years (31.3% of children abusers and 29.2% of adult abusers). Regarding marital status, the majority of abusers were married (for all participants 78.7%, for children 77.7%, and for adults 81.3%). Abusers commonly had a high school or equivalent degree (41.0%) and were employed (39.3%).

**Table 3:** Perpetrator Information (n = 178)

	Children		Adults		All	
	n	%	n	%	n	%
Relation to the victim						
Father	46	35.4	7	14.6	53	29.8
Mother	43	33.1	2	4.2	45	25.3
Both parents	26	20.0	1	2.1	27	15.2
Other	15	11.5	38	79.2	53	29.8
Gender						
Male	53	40.8	36	75.0	89	50.0
Female	51	39.2	11	22.9	62	34.8
Both	26	20.0	1	2.1	27	15.2
Age group						
0–18 years	3	2.3	0	0.0	3	1.7
19–30 years	23	17.7	4	8.3	27	15.2
31–40 years	52	40.0	17	35.4	69	38.8
41–50 years	38	29.2	15	31.3	53	29.8
51–60 years	3	2.3	8	16.7	11	6.2
> 60 years	3	2.3	3	6.3	6	3.4
Unknown	8	6.2	1	2.1	9	5.1
Marital status						
Single	6	4.6	6	12.5	12	6.7
Married	101	77.7	39	81.3	140	78.7
Divorced	2	1.5	2	4.2	4	2.3
Widowed	5	3.9	0	0.0	5	2.8
Separated	4	3.1	0	0.0	4	2.3
Unknown	12	9.2	1	2.1	13	7.3
Educational level						
Illiterate	8	6.2	7	14.6	15	8.4
Elementary	22	16.9	6	12.5	28	15.7
Intermediate	19	14.6	11	22.9	30	16.9
High school or equivalent	59	45.4	14	29.2	73	41.0
University or equivalent	12	9.2	8	16.7	20	11.2
Unknown	10	7.7	2	4.2	12	6.7
Employment						
Employed	50	38.5	20	41.7	70	39.3
Student	4	3.1	0	0.0	4	2.3
Freelancers	1	0.8	3	6.3	4	2.3
Housewife	50	38.5	8	16.7	58	32.6
Retired	8	6.2	6	12.5	14	7.9
Unemployed	7	5.4	9	18.8	16	9.0
Unknown	10	7.7	2	4.2	12	6.7
Health condition						
Well	103	79.2	37	77.1	140	78.7
Chronic illness	9	6.9	3	6.3	12	6.7
Disability	3	2.3	2	4.2	5	2.8
Addicted	4	3.1	4	8.3	8	4.5
Unknown	11	8.5	2	4.2	13	7.3

### Probability of Victim Final Status, Adults vs. Children, Gender, and Previous Abuse

Abused adults were more likely cared for by people other than their parents (OR = 41.63, 95% CI 14.82–116.97) relative to those who were cared for by both parents (Table 4). Those who were cared for by one parent were more likely to have a previous abuse than those who were cared for by both parents (OR = 4.11, 95% CI 1.43–11.81). In addition, caregivers who were other than parents were more likely to abuse males than females compared to those who were cared for by both parents (OR = 2.16, 95% CI 1.03–4.50). Female perpetrators were less likely to abuse adults than their male counterparts (OR = 0.32, 95% CI 0.15–0.69), and less likely than males to abuse victims with a previous abuse (OR = 0.23, 95% CI 0.10–0.56). Perpetrators who were 51 years and older were more likely to abuse adults than those of younger age groups (31–40 years) (OR = 5.61, 95% CI 1.80–17.46). Moreover, victims who were previously abused were more likely to have complications than those who were never abused (OR = 3.98, 95% CI 1.88–8.44).

**Table 4:** Associations of risk factors with victim's age, victim's gender, victim's final status and previous abuse

	Victim's age			Victim's gender			Victim's final status			Previously abused		
	Adult vs. child			Male vs. female			Complications vs. none			Yes vs. no		
	OR	(95% CI)	*p*-value	OR	(95% CI)	*p*-value	OR	(95% CI)	*p*-value	OR	(95% CI)	*p*-value
Caregivers			< 0.0001*			0.048*			0.664			0.015*
Both parents	1.00			1.00			1.00			1.00		
One parent	1.89	(0.73–4.92)		0.72	(0.34–1.54)		0.70	(0.30–1.61)		4.11	(1.43–11.81)	
Other	41.63	(14.82–116.97)		2.16	(1.03–4.50)		0.81	(0.37–1.76)		2.06	(0.88–4.83)	
Perpetrator gender			0.004*			0.764			0.166			< 0.0001*
Male	1.00			1.00			1.00			1.00		
Female	0.32	(0.15–0.69)		1.11	(0.58–2.12)		1.66	(0.81–3.42)		0.23	(0.10–0.56)	
Perpetrator age group			0.005*			0.841			0.320			0.307
0–30 years	0.47	(0.14–1.54)		1.40	(0.57–3.23)		1.83	(0.70–4.80)		1.24	(0.48–3.21)	
31–40 years	1.00			1.00			1.00			1.00		
41–50 years	1.21	(0.54–2.72)		1.20	(0.58–2.47)		0.94	(0.42–2.10)		1.21	(0.53–2.755)	
> 51 years	5.61	(1.80–17.46)		0.85	(0.28–2.56)		0.52	(0.15–1.80)		7.74	(0.93–64.73)	
Previously abused			0.185			0.321			< 0.0001*	NA		
Yes	1.69	(0.78–3.70)		1.40	(0.72–2.71)		3.98	(1.88–8.44)				
No	1.00			1.00			1.00					

*OR* Odds ratio, *CI* confidence intervals, *NA* not applicable

*Significant at p-value < 0.05 level

## Discussion

This study identified the prevalence of people with disabilities among reported abuse and the victims’ associated risk factors for abuse among people with disabilities in Saudi Arabia. Disabled individuals comprised 1.4% of all reported cases of abuse during the period 2017–2022. The highest prevalence rate was observed in 2017 (3.9%). The trend of people with disabilities among abused cases in this study has gradually decreased over the last 6 years, reaching its lowest prevalence rate of 0.8 in 2022. On the contrary, a study in Taiwan analyzed a 4-year trend (2006–2009) of reported abuse among people with disabilities and showed a significant increase in the rates of violence against disabled individuals [21]. Saudi Arabia has initiated multiple efforts to protect individuals with disabilities against different forms of abuse by developing regulations and policies such as the Law of Protection from Abuse, Disability Welfare Law, and Disability Rights Law. Together, they provide essential shelter along with social, psychological, and healthcare services. Additionally, legal steps are taken to prosecute and penalize those responsible for abuse.

Although the proportion of abused disabled individuals has declined, there was a slight increase in 2021, which might be attributed to the COVID-19 pandemic, which led to a period of isolation, quarantine, and lockdown. In times of crisis, reports of violence are often linked to economic instability and high-stress environments [22]. During the COVID-19 pandemic period, victims were forced to be locked down with their perpetrators, intensifying domestic violence, especially among females, and children [23]. In addition, the limited availability and accessibility of services could have hindered support for violence during a pandemic. Circumstances during the pandemic aggravated the risk of abuse and neglect due to family dynamics and the wider social environment [24]. Loss of income contributes to stress and anxiety and increases the likelihood of domestic abuse against children [25].

In our results, violence against people with disabilities was more common among children than adults in Saudi Arabia. A study in the United States had similar findings; children were more likely to be exposed to violence than adults [26–28]. Children are considered a vulnerable population and are more susceptible to abuse from the adults responsible for caring for them; therefore, they are more prone to abuse compared to adults [29]. Female perpetrators are less likely to abuse adults than male perpetrators. This indicates that males tend to abuse adults, whereas females tend to abuse children. Similar observations were made in a Canadian cohort; females tended to abuse younger children more than their male counterparts [30]. We also found that caregivers who are other than parents are more likely to abuse adults than children when compared to both parents. These results might be due to the nature of caregiving in the studied sample, since 73.6% of children were cared for by both parents and 60.0% of adults were cared for by a husband/wife or others. In addition, the older the perpetrator’s age the higher the probability of abusing adults; younger perpetrators were more prevalent among children. This might be because of the age difference between parents who abuse their children and husbands/wives who abuse their partners.

Regarding the gender of the victims, males and females were similarly abused in this study; females had a slightly higher violence proportion among the adult subgroup, whereas males had a higher violence proportion among the children group. According to the general disability statistics in Saudi Arabia, disabled males (57.5%) have a slightly higher presentation in the general population than females [19]. Studies in the United States and Denmark have shown that disabled females are more likely to experience sexual violence than males with disabilities; however, males with disabilities are more likely to experience physical violence [13, 31]. In a study in the United Kingdom, men with disabilities were more likely to be victims of physical and non-domestic violence and women were much more likely to be victims of sexual and domestic violence [12]. Moreover, according to a systematic review, women with severe mental disabilities are at a higher risk of experiencing violence [12]. However, no gender-related differences were found in the studies from Taiwan and Italy [21, 32]. Additionally, in another study, there was no gender difference in any type of abuse, except for sexual abuse, which was more common among females [33]. It is noteworthy, that although we found a significant association between caregivers (parents vs. others) and the gender of the victim, it is unclear what sort of association exists because no other factors were significant.

Less than half of the abuse cases involved victims who experienced previous abuse. The nature of the abuse was mostly neglect or physical abuse, similar to the nature of the previous abuse. Yet another study in South Africa indicated that structural violence ranked highest, followed by psychological physical, and sexual violence ranked the lowest [34]. In many instances, abuse is not an isolated or one-time event but rather a recurrent one, which might be a repetitive incident of the same nature [28]. Multiple studies have confirmed that violence is a recurrent issue, with an average of six incidents per victim with disabilities; some participants reported up to 14 incidences of violence [34, 35]. Therefore, in cases having previously identified abuse, a proactive approach is needed to prevent recurrent violence. These efforts might include counseling and home visits to support the victim, with ways to identify and report abuse, if needed. Looking closer at factors associated with recurrent abuse, we found that those who were cared for by one parent were more likely to have experienced previous abuse than those who were cared for by both parents. Similar results were reported in a Saudi study that identified violence among children; participants who lived with a single parent were more likely to experience violence due to a lack of protection and safety [36]. In our study, female perpetrators were less likely to abuse previously abused individuals. Similar findings were observed in other studies; male perpetrators were more likely to repeat abuse [37, 38]. Moreover, victims who were previously abused were more likely to experience complications than those who had never been abused. A cross-sectional study also indicated that violence was associated with worse health status regardless of gender [33]. This demonstrates the seriousness of violence and the long-term effects that could impact victims and society. However, we found no association between the perpetrator’s gender and the victim’s final status.

Most cases of violence were reported by healthcare institution members or family members, and only 7% of cases were reported by the victims themselves. Individuals with disabilities might have difficulties seeking medical care without the help and support of their caregivers because of communication barriers, transportation barriers, and physical environment challenges. For instance, mobility impairments may hinder an individual’s ability to flee when physically or sexually abused. Sensory-impaired individuals may be unable to communicate about the abuse, especially when the abuser is interpreting [26]. This could also highlight the power imbalance or dominance between people with disabilities and their caregivers [39]. Moreover, most violence cases in this study were among unemployed or housewife victims, which indicated that the abused victim might be dependent on others for support, leaving them in a more vulnerable position, especially if the abuser or perpetrator is the provider (spouse or parent) [40]. In another study of Saudi children, parents were the most common perpetrators, followed by siblings [36], which indicates that power can be a contributing factor in abuse in general and specifically among the disabled.

Our study has several limitations. First, the reported incidents of violence in the registered hospitals and healthcare centers may not have captured true incidents. In addition, most abuse cases are not formally disclosed, and the reporting of incidents through other means, such as helplines, police, or others, might not be included in the NFSP database. The data were derived from registered centers. In a survey conducted among abused people with disabilities, only 37.3% reported this to authorities [11]. Moreover, children with disabilities may also lack communication skills. Second, the data lacked important variables such as the type of disability, which can provide a better understanding of the issues. Improvements in the case registration form are necessary to gather specific information systematically and ensure data accuracy. Third, although the number of cases of disability was low, this prevented further statistical analyses in which controlling for confounders could have been considered. Finally, the quantitative method applied in this study might help to understand the distribution and characteristics of the problem but not a definitive cause, since it did not consider human behavior and other important perceptions [41].

Although these limitations may have hindered the study, its strengths include the population representation of abused and people with disability. Since the data were obtained from a national registry for violence, they provided the most available results on violence against people with disabilities in Saudi Arabia. This is particularly important because there is limited literature and data on this topic. Most studies regarding this issue have been conducted in the United States and the United Kingdom [9]. The results of this study provide a broader perspective on this issue for stakeholders and policymakers. Defining the issue of violence among this population is one step in the public health approach to violence prevention.

## Conclusions

This study helps elucidate the significant issue of disability prevalence among individuals who have experienced abuse in the general population of Saudi Arabia. These findings underscore the urgent need for targeted interventions and support systems to address the intersection between abuse and disability. By acknowledging and addressing the unique challenges faced by abused individuals with disabilities, policymakers and stakeholders can foster more inclusive and equitable communities. Moreover, this study highlights the importance of comprehensive data collection and analysis to inform evidence-based strategies aimed at preventing violence and improving survivor well-being. Further studies should consider qualitative methods to better understand the scope of this issue in this population. These studies could focus on exploring the lived experiences of individuals with disabilities regarding barriers to accessing support services for abuse, communication barriers, stigma, and institutional challenges that impact their ability to seek assistance and access resources in instances of abuse. In addition, cross-sectional surveys would be valuable to assess the prevalence of abuse among people with disabilities in the community.

## Data Availability

The data that support the findings of this study are available from the National Family Safety Program but restrictions apply to the availability of these data, which were used under license for the current study, and so are not publicly available. Data are however available from the authors upon reasonable request and with permission from the National Family Safety Program, and King Abdullah International Research Center.
